# Transmission of Multiple HIV-1 Subtype C Transmitted/founder Viruses into the Same Recipients Was not Determined by Modest Phenotypic Differences

**DOI:** 10.1038/srep38130

**Published:** 2016-12-02

**Authors:** Hongshuo Song, Bhavna Hora, Elena E. Giorgi, Amit Kumar, Fangping Cai, Tanmoy Bhattacharya, Alan S. Perelson, Feng Gao

**Affiliations:** 1 Department of Medicine, Duke University Medical Center, Durham, NC 27710, USA; 2 Theoretical Division, Los Alamos National Laboratory, Los Alamos, NM 87545, USA; 3 National Engineering Laboratory for AIDS Vaccine, School of Life Sciences, Jilin University, Changchun 130012, China

## Abstract

A severe bottleneck exists during HIV-1 mucosal transmission. However, viral properties that determine HIV-1 transmissibility are not fully elucidated. We identified multiple transmitted/founder (T/F) viruses in six HIV-1-infected subjects by analyzing whole genome sequences. Comparison of biological phenotypes of different T/F viruses from the same individual allowed us to more precisely identify critical determinants for viral transmissibility since they were transmitted under similar conditions. All T/F viruses used coreceptor CCR5, while no T/F viruses used CXCR4 or GPR15. However, the efficiency for different T/F viruses from the same individual to use CCR5 was significantly variable, and the differences were even more significant for usage of coreceptors FPRL1, CCR3 and APJ. Resistance to IFN-α was also different between T/F viruses in 2 of 3 individuals. The relative fitness between T/F viruses from the same subject was highly variable (2–6%). Importantly, the levels of coreceptor usage efficiency, resistance to IFN-α and viral fitness were not associated with proportions of T/F viruses in each individual during acute infection. Our results show that the modest but significant differences in coreceptor usage efficiency, IFN-α sensitivity and viral fitness each alone may not play a critical role in HIV-1 transmission.

HIV-1 mucosal transmission is associated with a stringent population bottleneck that remains poorly understood. Despite the highly diverse quasispecies population in HIV-1-infected donors, about 60–80% of HIV-1 infections through mucosal transmission are established by a single transmitted/founder (T/F) virus^1^,^2^,^3^,^4^,^5^. Some evidence suggests that HIV-1 transmission is not entirely a stochastic process in which all replication competent viruses have an equal probability to be transmitted, but rather that certain genetic and phenotypic traits, which can facilitate viral transmission, are selected^6^. Genetic comparisons of the *env* sequences of T/F viruses showed shorter variable loops and fewer potential N-linked glycosylation sites for subtype A and C T/F viruses^3^,^7^,^8^,^9^, but not for subtype B T/F viruses compared to the donor or chronic viruses^8^,^10^,^11^. Comparison of the subtype B T/F viruses and chronic viruses identified some signatures in T/F *env* sequences^12^,^13^. Biological studies with infectious molecular clones (IMCs) representing unambiguously inferred full-length T/F HIV-1 genomes showed that T/F viruses replicate with high efficiency in primary CD4 + T cells, but less efficiently in monocyte-derived macrophages^2^,^14^,^15^. Recent comprehensive phenotypic comparisons between the T/F and chronic viruses demonstrated that T/F viruses have enhanced infectivity, incorporate higher Env content per viral particle, bind dendritic cells more efficiently and are relatively more resistant to inhibition by IFN-α than chronic viruses^16^,^17^.

Despite the severe population bottleneck, around 20–40% of mucosal HIV-1 transmissions are established by multiple HIV-1 T/F viruses^1^,^2^,^3^,^4^,^5^. The mechanisms for transmission of multiple HIV-1 variants into the same subjects are still unknown. Statistical analysis of cases of multiple HIV-1 transmissions through the heterosexual route revealed that the number of transmitted viruses does not follow a Poisson distribution, suggesting that the transmission of multiple T/F viruses are not independent events with low probability^4^. Therefore, it has been hypothesized that, during transmission of multiple HIV-1 variants, either the transmission rate is transiently increased due to certain cofactors, such as inflammatory genital infections that may reduce the transmission barrier by destroying the intact mucosa or increasing the availability of activated CD4 + T cells, or that transmission of multiple variants are linked events, such as through a multiply-infected cell or viral aggregates^3^,^4^. Regardless of the mechanisms involved, the fact that different HIV-1 variants can simultaneously cross the mucosal barrier in the same host and establish productive infection imply that these T/F viruses may have similar transmission efficiency. However, little is known to date regarding whether multiple T/F viruses in the same HIV-1-infected individual share the same phenotypic properties. Addressing this question would provide unique insight into critical viral properties associated with HIV-1 transmissibility and help to better understand potential mechanisms of HIV-1 transmission.

We have identified six individuals who were infected with two or more subtype C T/F viruses. To investigate if multiple T/F viruses in each individual have distinct biological phenotypes, we compared their coreceptor tropism, sensitivity to IFN-α inhibition and replication fitness. Our results demonstrated that T/F viruses in the same infected individual have different coreceptor usage efficiencies, resisted IFN-α at different levels and had different fitness. These observed differences indicate that those phenotypic properties cannot solely predict the transmissibility of HIV-1.

## Results

### Identification of multiple T/F whole genome sequences from the same individuals

Two or more T/F viruses were identified in six subjects by analyzing nearly full-length genome sequences (in two overlapping halves) generated by SGA from the first HIV-1 RNA positive plasma samples (at or before Fiebig stage III) in the CHAVI001 acute infection cohort (Table 1). The analysis of both halves of viral genomes using the Poisson model^18^ on each lineage and then combining the estimates as described in the methods showed that the first HIV-1 RNA positive samples were collected between 9 and 19 days after transmission for these six individuals (Table 1). In all of our time estimates, whether the infection was caused by single or multiple T/F viruses, we stated a 95% confidence interval whose width depended on the sample size (the number of sequences for analysis) (Supplementary Table 1). Within that time window, our model cannot confidently distinguish whether multiple genetically distinct T/F viruses infected the host at the same time or a few days apart. In most cases, estimates and confidence intervals calculated for different T/F virus lineages within the same individual overlapped. Goodness of fit p-values, which measured the probability that the overall Hamming distance (HD) distribution differed from the expected Poisson form, were non-significant after multiple-testing correction. Both elements indicated that different T/F virus lineages could have evolved at the same time. The one exception was the CH1754a lineage in the 5′-half genome, which seemed much more homogeneous than the other T/F lineages, indicating a possibility that CH1754a T/F virus was a later infection than the other T/F virus lineages causing the overall HD distribution to diverge from a Poisson form (Supplementary Table 1).

**Figure 1:**
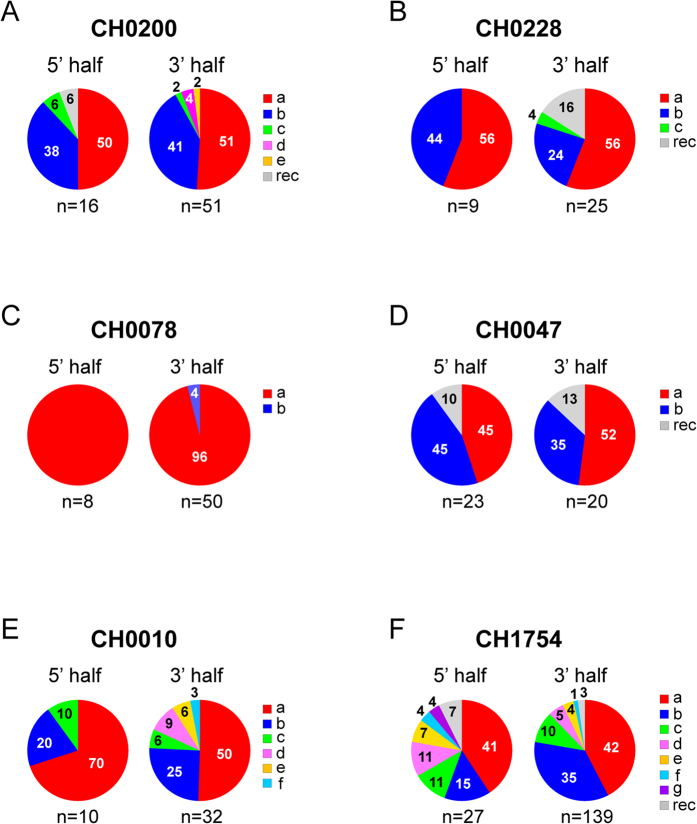
Percentage of T/F virus populations in each HIV-1-infected individual. The number of T/F viruses and their percentage were determined based on the 5′- and 3′-half viral genomes at the screening time point in subjects CH0200 (**A**), CH0228 (**B**), CH0078 (**C**), CH0047 (**D**), CH0010 (**E**) and CH1754 (**F**). Different T/F sequences were indicated by different colors and recombinants were shown as gray. The numbers on the pie charts indicate the percentage of each T/F virus as well as the recombinants. The number of SGA sequences (n) for 5′- and 3′-half genomes in each individual is indicated under the pie chart. The number included those from both half genome sequences and NFLG sequences for CH0200, and both long and short 3′-half genome sequences for CH0228.

Between two and seven T/F viruses were identified in each of the six subjects (Table 1 and Fig. 1). The proportions of T/F viruses in each individual were highly variable, with major T/F viruses as high as 96% in CH0078 and minor T/F viruses as low as 1% in CH1754 (Fig. 1). The highly unequal proportions of T/F viruses at acute infection suggest different replication advantages among T/F viruses in the same HIV-1-infected individuals.

### Different efficiencies in use of various coreceptors by T/F viruses from the same individual

**Figure 2:**
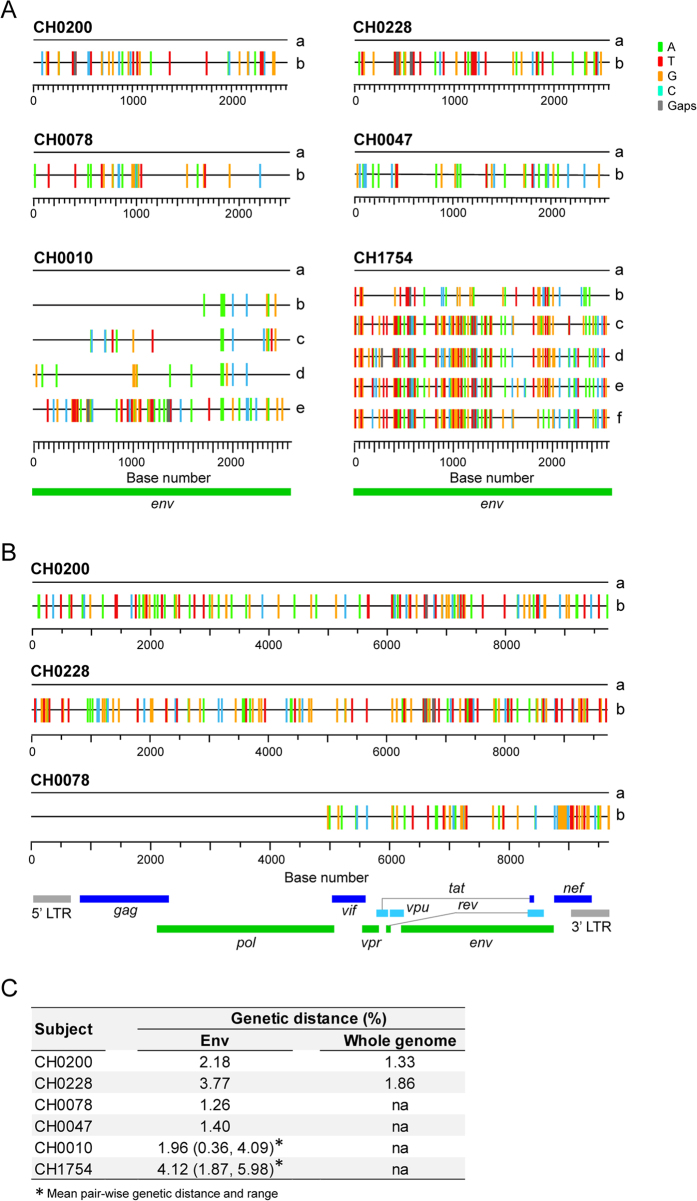
Genetic diversity among different T/F virus sequences in the same individuals. Highlighter plots (http://www.hiv.lanl.gov/content/sequence/HIGHLIGHT/highlighter_top.html?choice=mismatches) showed the locations of the base differences in the *env* gene (**A**) and the whole genome (**B**), compared to the most dominant T/Fa virus in each individual. Pairwise genetic distances among different T/F viruses in the *env* gene and the whole genome in each individual are shown (**C**).

**Figure 3:**
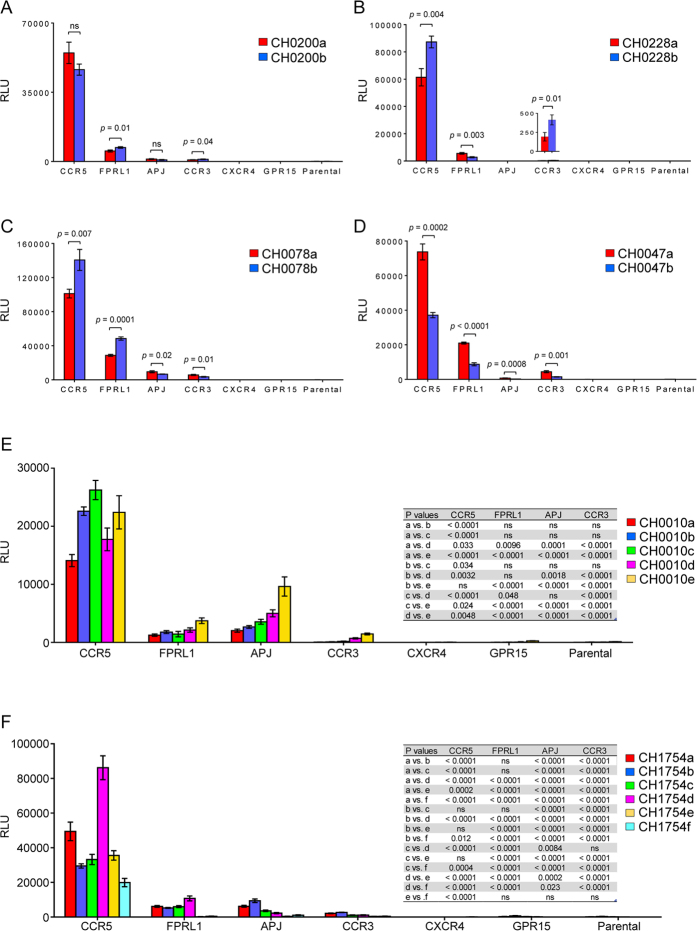
Comparison of coreceptor usage among different T/F viruses from the same HIV-1-infected individual. Same amount of T/F Env-pseudoviruses (200 TCID_50_) was used to infect NP-2 cell lines expressing CD4 together with one of other coreceptors (CCR5, CXCR4, FPRL1, APJ, CCR3 or GPR5). The parental NP-2 cell line expressing CD4 but not any coreceptor was used as a control. The luciferase activity was measured 3 days post infection. The positive virus replication was defined if a virus gave luciferase activity three times above its background luciferase activity in the NP-2 parental cell line. All infections were performed in triplicates.

Since proportions of different T/F viruses were highly variable in the same HIV-1 infected individual, one important question was whether they had different efficiencies in coreceptor usage that led to the disproportional expansion of different T/F viruses early after transmission. To address this question, we cloned all the *env* genes for which the T/F sequences could be reliably inferred with sufficient number of sequences. Two T/F *env* clones (a and b) were generated for CH0200, CH0228, CH0078 and CH0047, while five (a through e) and six (a through f) *env* clones were made for CH0010 and CH1754, respectively (Fig. 2A). The genetic diversity among the T/F *env* genes in each individual ranged from 1.26% (between two T/F *env* genes in CH0078) to 4.12% (among six T/F *env* genes in CH1754) (Fig. 2C). Env pseudoviruses were generated by cotransfection with the pNL43-ΔEnv-vpr^+^-luc^+^ clone that expresses luciferase upon infection and were used to infect a panel of NP-2 cell lines that express CD4 and one of two major coreceptors (CCR5 or CXCR4) or other commonly used coreceptors (FPRL1, APJ, CCR3 or GPR15)^19^,^20^,^21^,^22^. Consistent with previous reports^1^, all 19 T/F Env pseudoviruses were CCR5 tropic, while none of them used CXCR4 as coreceptor (Fig. 3). We also found that they all used coreceptors FPRL1 and CCR3, and 17 of 19 Env pseudoviruses, i.e., all except the two T/F viruses from CH0228, used APJ for entry, although the efficiency of these alternative coreceptors usage is much lower than of CCR5 usage (Fig. 3). None of the Env pseudoviruses infected GPR15 + cells.

Next, we investigated whether there was a difference in coreceptor usage efficiency between the T/F viruses from the same individual. Although all Env pseudoviruses used CCR5, the levels of infection among different T/F viruses were significantly different in 5 out of 6 individuals. Significant differences in use of the CCR5 coreceptor were found between the *env* genes of the two T/F from CH0228 (*p* = 0.004, two-tailed t-test), CH0078 (*p* = 0.007, two-tailed t-test) and CH0047 (*p* = 0.0002, two-tailed t-test), while no difference was observed between the two T/F *env* genes from CH0200 (Fig. 3A–D). The five T/F Env pseudoviruses from CH0010 significantly differed in infectivity (*p* < 0.0001, One-way ANOVA test), except for the comparison between CH0010b and CH0010e (Fig. 3E). When the six T/F Env pseudoviruses from CH1754 were compared, no differences in infectivity were observed among CH1754b, CH1754c and CH1754e, while all other T/F viruses were significantly different (*p* ≤ 0.012, One-way ANOVA test; Fig. 3F). The highest difference in infectivity in CCR5 + NP2 cells was observed between CH1754d and CH1754f (4.3 folds; *p* < 0.0001, One-way ANOVA test). These results showed that significant, but moderate, differences (<5 folds) in CCR5 usage were often observed among T/F viruses in the same infected subject.

Although T/F Env pseudoviruses also used other alternative coreceptors at much lower levels compared to CCR5, the infectivity of the various T/F from the same subjects varied much more in cells expressing those coreceptors than in CCR5 expressing cells (Fig. 3). CH1754e used CCR5 less efficiently than CH1754a and CH1754d, more efficiently than CH1754f, and similar to CH1754b and CH1754c (Fig. 3F). However, on average it was 66.5-fold less efficient in infecting FPRL1 + cells than CH1754d (*p* < 0.0001, One-way ANOVA test), 21.9-fold less efficient than CH1754b to infect APJ + cells (*p* < 0.0001, One-way ANOVA test), and 6.9-fold less efficient than CH1754b to infect CCR3 + cells (*p* < 0.0001, One-way ANOVA test). CH0010a used CCR5 1.6-fold less efficiently than CH0010e, but was 19.8-fold less efficient in infecting CCR3 + cells than CH0010e (*p* < 0.0001, One-way ANOVA test) (Fig. 3E). Various levels of differences in alternative coreceptor usage were also observed for different T/F viruses in four other individuals, although the differences were comparatively smaller (Fig. 3A–D). Taken together, these results showed that different T/F Envs from the same subject use the same coreceptor at differing efficiencies, and the efficiency in using CCR5 was not indicative for usage efficiencies of other coreceptors.

### Various sensitivities to IFN-α inhibition among T/F viruses from the same subject

**Figure 4:**
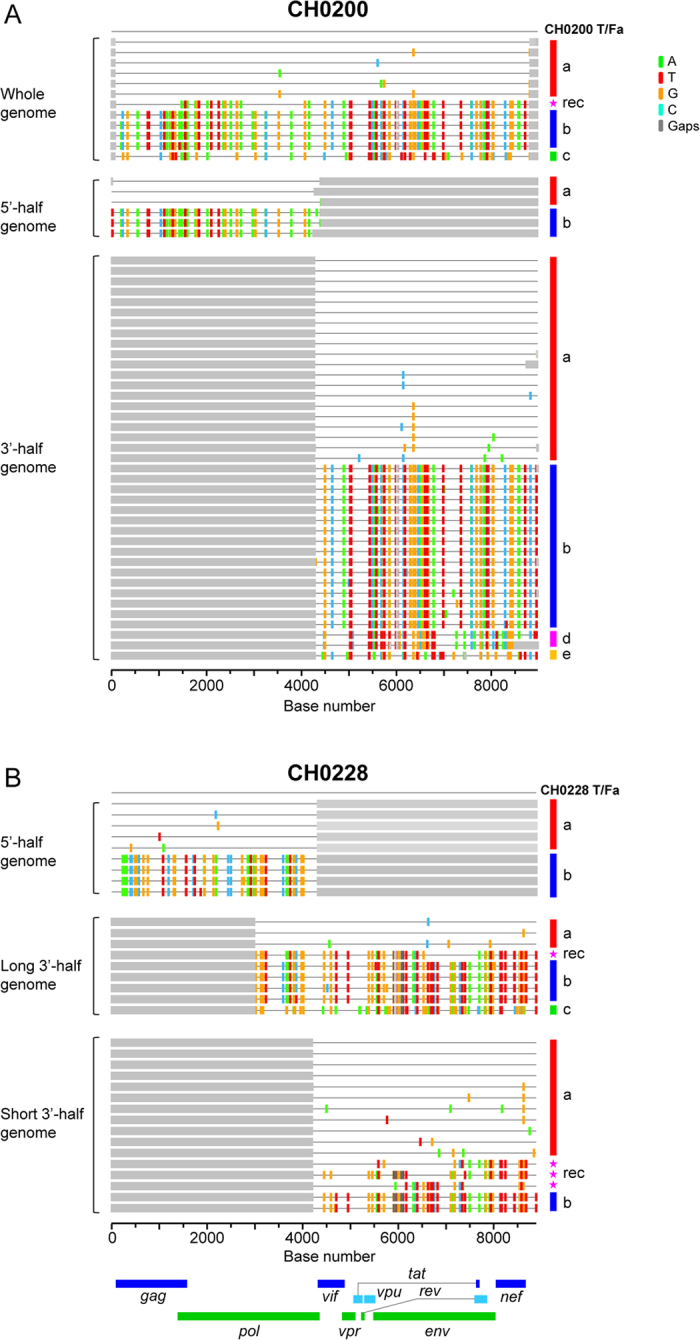
Identification of matching 5′- and 3′- half genomes from the same T/F virus in each individual. Highlighter plots identified the matching 5′- and 3′- half genomes from the same T/F virus by using NFLG genome sequences in CH0200 (**A**) and long overlapping sequences (~1000 bp) in CH0228 (**B**). The T/Fa and T/Fb sequences that were identical or highly similar in NFLG and both half genome sequences (CH0200) or in the overlapping region in the middle of HIV-1 genomes (CH0228) are indicated by red and blue bars, respectively.

Recent studies showed that subtype B T/F viruses were relatively more resistant to IFN-α than chronic viruses or matched 6-month viruses from the same infected individuals, while generally no significant differences were observed for subtype C viruses^17^,^23^,^24^. To determine whether T/F viruses from the same infected individual resist IFN-α at different levels, we synthesized T/F genomes and constructed infectious molecular clones (IMCs) to represent the T/F viruses for which the T/F whole genome sequences could be reliably inferred (Fig. 2B). To accurately match the 5′- and 3′-half genome sequences for each whole T/F genome in CH0200, we successfully obtained 12 near full length genome (NFLG) sequences by SGA. Highlighter plot analysis of all those sequences together showed that CH0200a and CH0200b sequences from 5′- or 3′-half genome were identical or highly similar to CH0200a and CH0200b NFLG sequences (Fig. 4A). Phylogenetic tree analysis of both half genome sequences confirmed that CH0200a and CH0200b from NFLG or half genome sequences were indistinguishable (Supplementary Fig. 1). Thus, both CH0200a and CH0200b 5′- and 3′ half genome sequences could be unequivocally linked together based on the NFLG sequences. We were unable to obtain NFLG sequences by SGA for the viruses in CH0228. Instead, we successfully amplified nine long 3′-half genome SGA sequences, which overlapped with the 5′-half genome sequences by ~1000 bp. Highlighter plot analysis of the overlapping region showed that both CH0228a and CH0228b from 5′- and 3′-half genome sequences were identical or highly similar at this overlapping region and the sequences from both long and short 3′-half genome sequences are indistinguishable (Fig. 4B). Phylogenetic tree analysis of the overlapping sequences confirmed these relationships (Supplementary Fig. 2). Thus, both analyses allowed for unambiguous identification of the correct 5′- and 3′-half genome sequences derived from the same T/F genome in CH0228 based on the overlapping region sequences. The missing LTR region for each T/F virus in the two overlapping half genome sequences was amplified independently as previously described^14^,^16^,^25^. Two T/F IMCs (a and b) were generated for subjects CH0200, CH0228 and CH0078 (Fig. 2B).

The screening samples were generally limited in volume since they were the first samples collected from all subjects. In addition, the 5′-half genome was more difficult to amplify than the 3′-half genome. Since we performed SGA analysis of 3′-half genome first, samples were often exhausted before obtaining a large number of SGAs for the 5′-half genome. This was why we only had relatively small numbers of SGAs for CH0078, CH0010 and CH0228. The minority T/F (CH0078b) viruses were only present at 4% of 3′-half genome sequences (50), we were not able to get enough SGAs to obtain sequences for this minority T/F in the 5′-half genome sequences before we exhausted the screening sample (Fig. 1C) and only one 5′-half genome derived from the CH0078b T/F virus was detected 4 weeks later^26^. To determine if the 3′-half genome could have any impact on biological phenotypes, two chimeras that shared the same 5′-half genome were generated and tested in this study since the full-length T/F sequences for the CH0078b T/F virus could not be reliably inferred (Fig. 2B).

**Figure 5:**
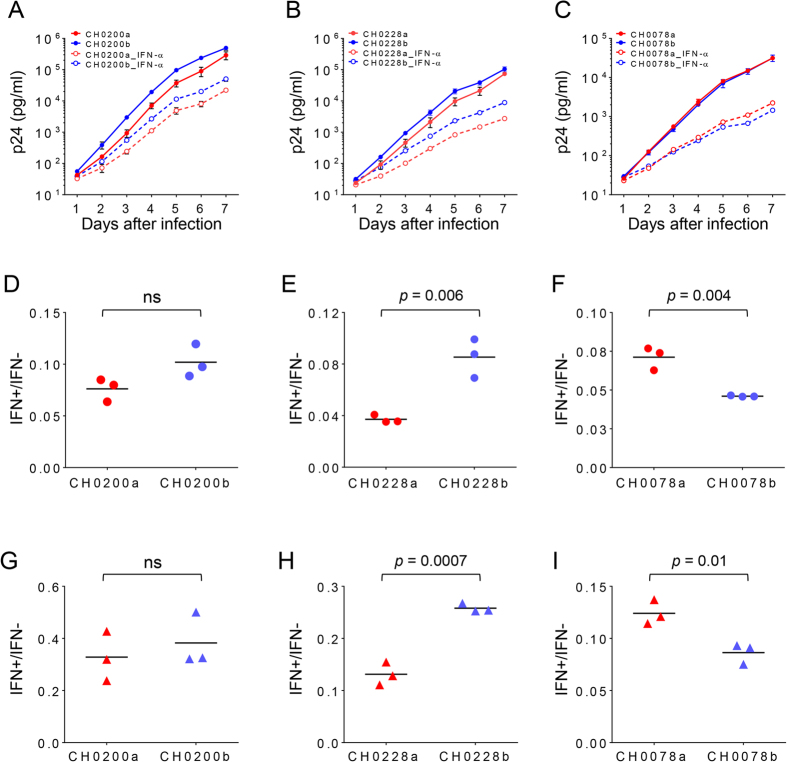
Sensitivity of different T/F viruses to IFN-α inhibition. The replication kinetics of each multiple T/F virus in the absence (solid lines) and presence of 500 U/ml IFN-α (dashed lines) from subjects CH0200 (**A**), CH0228 (**B**), CH0078 (**C**) were determined in primary CD4 T cells from a health donor C24. Activated primary CD4 + T cells from donor C24 were infected with equal amount (500 TCID_50_) of each virus. Viral replication was monitored by measuring the p24 concentration in the culture supernatant daily for 7 days. All infections were performed in triplicates. The resistance to IFN-α inhibition by different T/F viruses from CH0200 (**D**), CH0228 (**E**), CH0078 (**F**) was determined by comparing the ratio of viral p24 production in the presence and absence of IFN-α at day 7. The resistance to IFN-α inhibition by different T/F viruses from CH0200 (**G**), CH0228 (**H**) and CH0078 (**I**) was also determined in primary CD4 + T cells from another donor C29 at day 7.

Different T/F viruses from subjects CH0200, CH0228 and CH0078 had very similar replication kinetics in activated primary CD4 + T cells when each was cultured independently (Fig. 5A–C). All T/F viruses replicated at significantly lower levels in the presence of 500 U/ml IFN-α than in the absence of IFN-α (dashed lines in Fig. 5A–C). To compare the level of resistance to IFN-α for each virus, we determined the ratio of p24 concentrations in the culture with or without IFN-α at day 7 as previously reported^16^. In CH0228, the major T/F virus CH0228a was on average 2.3-fold more sensitive to IFN-α than a less predominant T/F virus CH0228b (*p* = 0.006, two-tailed t-test, Fig. 5E). But in CH0078, the major T/F virus CH0078a was on average 1.5-fold more resistant to IFN-α than the minor T/F virus CH0078b (*p* = 0.004, Fig. 5F). No significant difference was observed between the most predominant virus CH0200a and a less predominant virus CH0200b in CH0200 (Fig. 5D). Similar results were obtained with primary CD4 + T cells from a different donor (Fig. 5G–I).

### Various fitness differences among T/F viruses from the same subject

**Figure 6:**
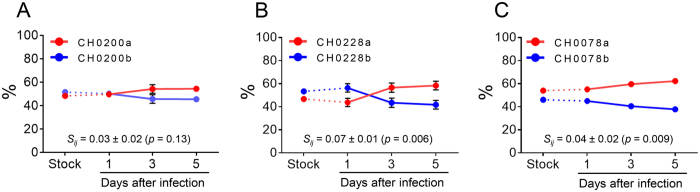
Comparison of viral fitness of different T/F viruses from the same HIV-1-infected individual. The relative fitness of different T/F viruses in the same individual was determined using the competition PASS fitness assay^27^,^29^. Activated primary CD4 + T cells were infected with viral stock mixture containing equal amount (5 ng p24) of each compared virus. The proportions of compared viruses in the culture supernatant were measured on days 1, 3 and 5. The major (red) and the minor or less predominant (blue) T/F viruses from CH0200 (**A**), CH0228 (**B**) and CH0078 (**C**) were compared.

We next sought to investigate whether different T/F viruses in the same individual had similar replication fitness. To more sensitively compare the fitness of T/F viruses, we performed competition PASS fitness assays, as previously described^27^,^28^,^29^,^30^. Two T/F viruses from CH0200, CH0228 or CH0078 were cultured together and the proportion of each virus in the culture was determined at days 1, 3 and 5 by PASS. The less predominant T/F virus CH0200b was nearly as fit as the major T/F virus CH0200a (relative fitness 3% ± 2% [mean of 3 replicates ± SE, see methods], p = 0.13) (Fig. 6A). The less predominant T/F virus CH0228b was 7% less fit than the major T/F virus CH0228a (p = 0.006) (Fig. 6B). Although the minor T/F virus in CH0078 accounted for only 4% of the viral population at the screening time point^26^, the major T/F virus was only a little more fit than the minor T/F virus in CH0078 (relative fitness 4% ± 2%, p = 0.009) (Fig. 6C). Overall, major T/F viruses were more fit than the less predominant or minor T/F viruses in three viruses but only in the cases of CH0228 and CH0078 was the difference statistically significant.

### No association between predominance of T/F viruses and phenotypes

We next determined whether the predominance of a T/F virus was associated with more efficient use of coreceptors, more resistance to IFN-α inhibition and better fitness in the same subject during acute infection. We found that the most predominant T/F viruses used CCR5 less efficiently than the less predominant or minor T/F viruses in CH0228, CH0078, and CH0010 (Fig. 3B,C,E and F) while the most predominant T/F virus used CCR5 more efficiently in CH0047 (Fig. 3D). In CH1754, the most predominant virus CH1754a used CCR5 significantly less efficiently than the minor T/F virus CH1754d (*p* < 0.0001, One-way ANOVA test), although with significant higher efficiency than all other four viruses (Fig. 3F). The predominant T/F virus was more resistant to IFN-α in CH0078, but less resistant to IFN-α than the minor T/F virus in CH0228. In the third subject CH0200, no significant difference in resistance to IFN-α was observed between the most predominant T/F virus and a less predominant one. The predominant T/F virus was generally more fit than the minor T/F virus in all three subjects (CH0200, CH0228 and CH0078). However, the major T/F virus was only 4% more fit in CH0078 compared to a 7% difference in fitness in CH0028, but the major CH0078a T/F virus dominated the minor CH0078b T/F virus (96% vs. 4%), while the CH0228a was a little more prevalent than the CH0228b (56% vs 24%). These results indicated that the proportions of T/F viruses in each HIV-1-infected individual were not determined by observed differences in efficiency in coreceptor usage, sensitivity to IFN-α or viral fitness alone at the acute infection stage.

## Discussion

Understanding the properties of T/F viruses will have important implications for HIV-1 vaccine development. Comparing the biological phenotypes among different T/F viruses from the same individuals infected with HIV-1 through mucosal transmission can reveal if some biological phenotypes determine HIV-1 transmission since these T/F viruses are likely transmitted under similar conditions. We compared biological phenotypes of different T/F viruses from each of six subjects and found significant differences in coreceptor usage efficiency, resistance to IFN-α and viral replication fitness among T/F viruses from the same individuals. These results indicate that transmission of different T/F viruses from a donor into a new recipient was possibly not determined solely by observed differences in CCR5 usage, alternative coreceptor usage, resistance to IFN-α, and viral replication fitness.

We found that the ability of different T/F Envs from the same individual to infect CCR5 + cells varied up to 4.3 fold, indicating that viruses with these levels of difference in CCR5 usage efficiencies could still all be efficiently transmitted into a new host. Previous studies have examined the usage of alternative coreceptors by HIV-1 isolates from acute/early infections^31^,^32^. However, little is known about the utilization of alternative coreceptors by T/F viruses and their potential roles in HIV-1 transmission. We found that the majority of the T/F Envs did not use CXCR4 or GPR15, but could use FPRL1, APJ and CCR3 for entry, although at reduced efficiencies compared to the use of CCR5. The usage of FPRL1, APJ and CCR3 differed by 20–67 folds between different T/F Envs from the same individuals (CH1754 and CH0010). These results showed that T/F Envs with significant differences in use of alternative coreceptors could again all be transmitted into the same recipient, suggesting that efficiency in use of these alternative coreceptors may also not be an important factor for HIV-1 transmission.

HIV/SIV can lead an interferon storm during acute infection^33^,^34^,^35^. Previous studies showed that subtype B T/F viruses were more resistant to IFN-α inhibition than chronic HIV-1 viruses from different individuals or matched 6-month viruses from the same infected individuals^16^,^17^. However, no statistical differences in IFN-α resistance were observed for subtype C T/F viruses compared to chronic virus or linked viruses from the donors quasispecies^16^,^24^, although two IMC-derived subtype C T/F viruses showed enhanced resistance than matched 6-month viruses after infection^17^. We now showed that modest differences (<3 folds) in IFN-α resistance were present among different T/F viruses from the same individuals infected with subtype C viruses. These data suggest that the observed differences in resistance to IFN-α among subtype C viruses, similar to those observed between acute and chronic viruses, may not be sufficient to explain the differential success of viruses after HIV-1 transmission.

The relationship between fitness and transmission of HIV-1 has been investigated based on partial viral genomes in previous studies^36^,^37^,^38^. Results were not conclusive since other parts of the viral genome can also affect viral fitness and play a critical role in transmission. Thus, the role of viral fitness in HIV-1 transmission remains unclear. A recent study on epidemiologically linked heterosexual transmission pairs showed that T/F virus did not exhibit higher replication capacity than the corresponding non-transmitted variants in the donors, which argued against the importance of viral replication fitness during transmission^24^. In our study, the complete genome sequence for each T/F viruses was unequivocally determined and free of RT-PCR artifacts since each T/F whole genome sequence was generated by the consensus of multiple SGA sequences. We found that the fitness differences were 3–7% between different T/F viruses in each of three individuals. All those individuals were infected through mucosal transmission based on the patient infection history. Thus, our results indicate that differences in viral replication fitness of this magnitude, as measured *in vitro*, do not affect HIV-1 transmission through the mucosal barrier. However, the study of additional cases is warranted to better characterize the role of replication fitness in HIV-1 transmission.

Similar differences in Env content, cell-free infectivity, dendritic cell interaction and IFN-α resistance were also observed between genetically much more divergent T/F and chronic viruses from different individuals^23^. In addition, no differences in CCR5 usage were found between T/F and chronic viruses from different individuals^23^,^39^. Thus, the subtle but significant differences in fitness and coreceptor usage between different T/F viruses in the same subject should represent the real differences among different T/F viruses. However, such differences were not likely to play an important role in transmission since different T/F viruses were transmitted at the same time. HIV-1 transmission may not be determined by any one of these biological characteristics alone, instead it is possible that it is governed by these viral properties in concert^23^. Of note, two CH0078 IMCs shared the same 5′-half CH0078a T/F genome because that 5′-half CH0078b T/F genome were not available. Thus, the differences in sensitivity to IFN-α inhibition and replication fitness in this case were only determined by 3′-half genome sequences. One limitation of this study is its relative small number of subjects. Future studies with a large sample size including subjects infected with other HIV-1 subtypes are warranted to confirm these observations.

Taken together, coreceptor usage efficiency, resistance to IFN-α, and replication fitness were different among multiple T/F viruses in the same individuals, suggesting that these observed differences each alone may not be sufficient to determine HIV-1 transmissibility. Moreover, the proportion of each T/F virus during acute infection was not associated to any of these biological phenotypes investigated in this study. This indicates that replication advantages of a T/F virus after transmission into a recipient may be determined by other host or viral factors. Whether higher levels of differences in these factors or other biological phenotypes are important to determine HIV-1 transmission remain to be studied.

## Methods

### Analysis of near full-length HIV-1 genome sequences

Six subjects who were infected with multiple T/F viruses were identified from the CHAVI001 acute infection cohort. All subjects were male and none were on antiretroviral therapy. All subjects were infected with subtype C viruses through heterosexual transmission. Written informed consent was obtained from all subjects and the study was approved by the Duke University Institutional Review Board. All methods were performed in accordance with the relevant guidelines and regulations. Viral RNA was extracted from plasma samples using the Qiagen EZ1 Virus mini kit on the BioRobot EZ1 (Qiagen, Valencia, CA) and used for cDNA synthesis using Superscript III Reverse Transcriptase (Life Technologies; Carlsbad, CA) and primers 1R3.B3R (5′-ACTACTTGAAGCACTCAAGGCAAGCTTTATTG-3′; nt 9611-9642 in HXB2) and 07Rev9 (5′-CTTCCTGCCATAGGAGATGCCTAA-3′; nt 5957-5980) for 3′- and 5′-half HIV-1 genome, respectively. Single genome amplification (SGA) was performed to obtain two overlapping half genomes as described^16^,^40^. For amplification of a long 3′-half genome (~6000 bp) of CH0228, the first round PCR was carried out with primers 07for5 (5′-GGDSTGCCCACACTAATGAT-3′; nt 3622-3641)and 2.R3.B6R (5′-TGAAGCACTCAAGGCAAGCTTTATTGAGGC-3′; nt 9636-9607); the second round PCR was done with primers IMC_For3 (5′-GAAAGCATAGTAATATGGGGAAAGACTCC-3′; nt 3681-3709) and Low2c 5′-TGAGGCTTAAGCAGTGGGTTCC-3′; nt 9591-9612). The PCR thermocycling conditions were as follows: one cycle at 94 °C for 2 min; 35 cycles of a denaturing step at 94 °C for 15 sec, an annealing step at 55 °C for 30 sec, an extension step at 68 °C for 6 min and 15 sec; and one cycle of an additional extension at 68 °C for 10 min.

For amplification of the near full length genome of CH0200, the first round PCR was carried out using primers Upper1A (5′-AGTGGCGCCCGAACAGG-3′; nt 634-650) and 2.R3.B6R, and the second round PCR was done with primers Upper2 (5′-CTCTCTCGACGCAGGACTCGGCTT-3′; nt 681–704) and LTRD (5′-CTGGAAAGTCCCCAGCGGAAAGTC-3′; nt 9460-9437). Two microliters of the first round PCR products were used for the second round PCR. The PCR thermocycling conditions were as follows: one cycle at 94 °C for 2 min; 10 cycles of a denaturing step at 94 °C for 15 sec, an annealing step at 55 °C for 30 sec, an extension step at 68 °C for 8 min; 20 cycles of a denaturing step at 94 °C for 15 sec, an annealing step at 55 °C for 30 sec, an extension step at 68 °C for 8 min with a incremental 20 sec for each successive cycle; and one cycle of an additional extension at 68 °C for 10 min.

SGA amplicons were sequenced directly by cycle sequencing and dye terminator methods using an ABI 3730xl genetic analyzer (Applied Biosystems, Foster City, CA). Individual sequences were assembled and edited using the Sequencher program 4.10 (Gene Codes, Ann Arbor, MI).

### Estimating days since infection

The days from infection were estimated using the Poisson model^18^ based on pairwise SGA sequence Hamming distances at screening calculated within each distinct lineage and then combined using the algorithm described below. Specifically, we divided each first-time point alignment into single-founder lineages. Sequences significantly enriched for hypermutation (p < 0.1 by Fisher exact test), detected recombinants, and sequences with ambiguous nucleotides were excluded from the analysis. Lineages with less than 3 sequences remaining were also excluded to avoid ambiguities in inferring the lineage consensus. For each of the remaining lineages, we calculated the Hamming distance (HD) of each sequence from the lineage consensus, which was taken to be the lineage T/F, and then merged all the HDs from the same alignment into one distribution. If all T/Fs evolved from the same time point, they were all equally fit and accumulated random mutations at the same rate, then the combined HDs should follow a Poisson distribution. We estimated the parameter of this Poisson distribution and used it to obtain the time since infection^18^. For each subject, time estimates from the two genome-halves were then averaged using a harmonic mean, which minimizes the asymptotic sampling variance.

### Generation of infectious molecular clones

The full-length genomes for T/F viruses were inferred as described in previous studies^14^,^25^. Infectious molecular clones (IMCs) were chemically synthesized and constructed for different T/F viruses from three subjects (CH0200, CH0228 and CH0078) as previously described^2^,^14^. Among them, CH0200a, CH0228a and CH0228b were reported in a previous study^16^. The viral stocks were generated by transfecting the IMCs into 293T cells as described previously^25^. The p24 concentrations of viral stocks were determined with the p24 ELISA kit (PerkinElmer, Waltham, MA) and TCID_50_ of viral stocks was determined on TZM-bl cells.

### Generation of Env pseudoviruses

The *env* gene of each T/F virus was amplified from the SGA-derived PCR amplicon of the 3′-half viral genome using the *Taq* High Fidelity polymerase (Invitrogen Life Technologies, Carlsbad, CA). The *env* amplicons were then gel-purified and ligated into the pcDNA3.1 vector using the pcDNA3.1 Directional TOPO Expression Kit (Invitrogen Life Technologies, Carlsbad, CA) as described previously^41^. All *env* clones were confirmed by sequencing. To produce Env-pseudoviruses, 6 μg of each *env* clone was co-transfected with 10 μg of pNL43-ΔEnv-vpr^+^-luc^+^ into 293T cells using the FuGENE6 transfection reagent (Roche Diagnostics; Indianapolis, IN). The culture supernatants containing the pseudoviruses were harvested 72 hours post transfection, aliquoted and stored at −80 °C until use. Infectious titers (TCID_50_) of pseudovirus stocks were determined on TZM-bl cells.

### Determination of coreceptor usage

The usage of coreceptors was determined on a panel of NP-2 cell lines expressing CD4 together with CCR5, CXCR4 or other G protein-coupled receptors (FPRL-1, APJ, CCR3 and GPR15)^31^,^42^. The parental NP-2 cell line expressing CD4 but not coreceptors was used as a control. NP-2 cell lines expressing different coreceptors were seeded into a 24-well plate one day before infection at a density of 1 × 10^5^ cells per well. On the next day, NP-2 cells were infected with same amount (200 TCID_50_) of each pseudovirus. After 4 hours of incubation at 37 °C, the infected cells were washed twice with complete culture medium and cultured with DMEM with 10% FBS at 37 °C for three days. Infected cells were lysed 72 hours post infection and the infectivity was assessed by measuring relative luciferase units (RLU) of lysed cells from each well. All experiments were performed in triplicates. Viral replication in each NP-2 coreceptor cell line was considered positive when the RLU value was three times above the background RLU value in the parental NP-2 cell line.

### IFN-α inhibition assay

Peripheral blood mononuclear cells (PBMCs) were obtained by leukopheresis from healthy donors as described previously^27^. Written consent was obtained from the donors and the study was approved by the Duke University Institutional Review Board. All methods were performed in accordance with the relevant guidelines and regulations. The CD4 + T cells were negatively selected from PBMCs with an auto MACS Pro Separator using the CD4 + T cell Isolation Kit II (Miltenyi Biotec, Auburn, CA). Before infection, purified CD4 + T cells were stimulated for 3 days in RPMI 1640 medium containing 10% fetal bovine serum (FBS), 32 U/ml interleukin2 (IL-2) (Advanced Biotechnologies, Columbia, MD), 0.2 mg/ml soluble anti-CD3 (eBioscience, San Diego, CA) and 0.2 mg/ml anti-CD28 (BD Bioscience, San Diego, CA). The stimulated CD4 + T cells were seeded into a 96-well U bottom plate (5 × 10^5^ cells per well) and infected with 500 TCID_50_ of each virus (m.o.i of 0.001). After incubation at 37 °C for 4 hours, the cells were washed 3 times with RPMI 1640 and then cultured in a 48-well plate with 500 μl of RPMI 1640 containing 10% FBS and 32 U/ml IL-2. Each virus was cultured in triplicates. Viral replication kinetics was monitored daily for 7 days by measuring p24 concentrations in the culture supernatant.

To determine virus sensitivity to IFN-α inhibition, stimulated CD4 + T cells were infected with each virus as described above. After washing, the infected cells were cultured in 500 μl of RPMI 1640 containing 10% FBS, 32 U/ml IL-2 and 500 U/ml IFN-α as previously described^16^. The viral replication in the presence of IFN-α was monitored daily for 7 days by measuring p24 concentrations in culture supernatants. On day 3, half of the culture medium in each well was exchanged with fresh medium containing 500 U/ml IFN-α. To compare the sensitivity to IFN-α inhibition, the ratio of viral p24 production in the presence and absence of IFN-α was calculated for each virus on day 7. All experiments were performed in triplicates.

### PASS fitness assay

Viral fitness was compared using a single-round competitive PASS fitness assay as we described previously^27^. In brief, purified CD4 + T cells were stimulated with soluble anti-CD3 and anti-CD28 for three days. After stimulation, 50 μl of cell suspension (1 × 10^6^) was seeded into each well of a 96-well U bottom plate and infected with the vial stock mixture containing equal amount of each compared virus (5 ng p24 per virus; ~0.001 m.o.i). After absorption at 37 °C for 4 hours, the cells were washed 3 times with RPMI 1640. The infected cells were then transferred into a 24-well plate and cultured with 600 μl of RPMI 1640 containing 10% FBS and 32 U/ml IL-2 for 5 days. The culture supernatant was harvested and replaced with fresh medium daily. The kinetics of virus replication was monitored by measuring the p24 concentration in the culture supernatant. All experiments were performed in triplicates.

The harvested culture supernatants were treated with RNase-free DNase to eliminate the residual plasmid DNA in viral stocks used for transfection. Viral RNA was then extracted from 200 μl of the treated supernatant using the PureLink Viral RNA/DNA Mini Kit (Invitrogen, Carlsbad, CA). RNA was eluted with 20 μl of RNase-free water. A total of 17 μl viral RNA was used for cDNA synthesis using the SuperScript III reverse transcriptase (Invitrogen, Carlsbad, CA) with viral specific primers. The cDNA was stored at −20 °C until use.

The parallel allele-specific sequencing (PASS) assay was performed as described previously^27^,^29^. The in-gel PCR reaction was performed in a PTC-200 Thermal Cycler using amplification primers specifically designed for viruses from each subject (CH0200 forward: 5′-TAGATCCCAAAATATAACAGACAATGCCAAGAC-3′ (nt 7040–7072 in HXB2) and CH0200 reverse: 5′Acry-CCCAAGAACCCAAGGAACACAGC-3′ (nt 7773–7795) for subject CH0200; CH0228 forward: 5′-TGCAATACCTCAGCCATAACACAA-3′ (nt 6810–6833) and CH0228 reverse: 5′Acry-TTCCAGAGCAGCCCCAAATC-3′ (nt 8006–8025) for subject CH0228; CH0078 forward: 5′-ATTTTTGTGCTCCAGCTGGTTATG-3′ (nt 6871–6894) and CH0078 reverse: 5′Acry-TTTCCTCCATCACGTGTCAATAGTAATCC-3′ (nt 7575–7603) for subject CH0078). The proportion of each virus in the culture supernatant was determined by annealing the sequencing primers to the PCR amplicons for single base extension (SBE) with fluorescent-labeled nucleotides specific for each compared virus (CH0200 seq: 5′-ACAGACAATGCCAAGACAATAATAGTACATCTT-3′ (nt 7056–7088) for subject CH0200; CH0228 seq: 5′-TGTCCAAAGGTCTCTTTTGACCCAAT-3′ (nt 6837–6862) for subject CH0228; CH0078 seq: 5′-GACAATGTCAAAACAATAATAGTCCA-3′ (nt 7059–7084) for subject CH0078^29^. The relative fitness (*s*_*ij*_) was determined as we previously described^27^.

### Statistical analysis

Values obtained from different T/F viruses were compared using a two-tailed t*-*test or one-way ANOVA test followed by multiple comparisons using the Prism 6.0 software. P values less than 0.05 were considered significant. Relative fitness differences from each replicate were analyzed as previously described^27^. In particular, these fitness differences were analyzed using a two-stage procedure^43^, assuming they were subject to two independent random measurement errors: a standard error (*σ*_*i*_ for the *i*^th^ replicate) in the determination of the fitness from each of the *n* replicates, arising both from Poisson fluctuations in the PASS assay and fluctuations in the viral growth over time [24], and a replicate-to-replicate variance (*σ*_*R*_) in the experimental setup. These two variances were separately estimated and added in quadrature to obtain a conservative^44^ estimate of the final standard error 
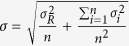
. Assuming that the *σ*_*R*_ are determined more accurately than *σ*_*R*_, we use the mean fitness difference along with *σ* in a t-test to calculate the significance of the fitness difference, with the degrees of freedom given by the one less than the number of replicates. We prefer this as being more conservative estimate than the Satterthwaite approximation which would multiply the degrees of freedom by *σ*^*4*^*/σ*_*R*_^*4*^ (ref. 45). To correct for the number of fitness comparisons being made in this study, we followed Bonferroni^46^ and reduced our threshold of significance from the nominal *p = 0.05* to *p = 0.05/n*_*comp*_, where *n*_*comp*_* = 3* denotes the number of comparisons.

All the calculations of fitness differences were done using the statistical package R^47^ and the nonlinear fits used the R package subplex^48^ and ucminf^49^.

## Additional Information

**How to cite this article**: Song, H. *et al*. Transmission of Multiple HIV-1 Subtype C Transmitted/founder Viruses into the Same Recipients Was not Determined by Modest Phenotypic Differences. *Sci. Rep.* **6**, 38130; doi: 10.1038/srep38130 (2016).

**Publisher's note:** Springer Nature remains neutral with regard to jurisdictional claims in published maps and institutional affiliations.

## Supplementary Material

Supplementary Information

## Figures and Tables

**Table 1:** Demographic characteristics of subjects infected with multiple T/F viruses.

Subject	Fiebig stage	Country	Year	Days from infection (95% CI)*	No. of T/F viruses	
					5′ half	3′ half
CH0200	I/II	Malawi	2007	11 (8, 13)	3	5
CH0228	III	Malawi	2007	19 (14, 24)	2	3
CH0078	I/II	South Africa	2007	11 (8, 14)	1	2
CH0047	III	Malawi	2007	19 (13, 24)	2	2
CH0010	I/II	Malawi	2006	9 (7, 12)	3	6
CH1754	III	Malawi	2010	14 (12, 15)	6	7

Days from infection were estimated taking the harmonic average of the estimates obtained using the two genome halves (see Supplementary Table 1).
