# Supplementary material for: Transmission of Multiple HIV-1 Subtype C Transmitted/founder Viruses into the Same Recipients Was not Determined by Modest Phenotypic Differences
Source: Sci Rep. 2016 Dec 2;6:38130. doi: 10.1038/srep38130 (PMC5133561; doi:10.1038/srep38130)
Supplement: Supplementary Information [file srep38130-s1.pdf]

Transmission of Multiple HIV-1 Transmitted/founder Viruses into the Same Recipients Was not Determined by Modest Phenotypic Differences

Subtitle: Disparate phenotypes of HIV-1 T/F viruses

Hongshuo Song[1], Bhavna Hora[1], Elena E Giorgi[2], Amit Kumar[1], Fangping Cai[1], Tanmoy Bhattacharya[2], Alan S Perelson[2] and Feng Gao[1,3*]

[1]Department of Medicine, Duke University Medical Center, Durham, NC 27710, USA
[2]Theoretical Division, Los Alamos National Laboratory, Los Alamos, NM 87545, USA
[3]National Engineering Laboratory for AIDS Vaccine, School of Life Sciences, Jilin University, Changchun 130012, China.

[*]Corresponding to:
Feng Gao, M.D.
Email: fgao@duke.edu

Supplementary Table 1. Estimation of days post infection for T/F viruses in each subject

Virus	3'-half genome				5'-half genome			
	T/F	No. of Sequences	Day post infection (95% CI)	p-value	T/F	No. of Sequences	Day post infection (95% CI)	p-value
CH0010	a	21	8 (5, 11)	0.744	a	7	17 (5, 28)	0.798
CH0010	b	13	11 (4, 18)	0.927				
CH0010	all	34	9 (7, 11)	0.825				
CH0200	a	24	13 (8, 18)	96	a	6	3 (0, 8)	0.63
CH0200	b	21	15 (11, 19)	82	b	6	10 (1, 19)	0.835
CH0200	all	45	15 (13, 17)	6	all	12	7 (4, 9)	0.653
CH0228	a	14	26 (16, 36)	0.245	a	5	20 (10, 29)	0.62
CH0228	b	6	15 (5, 25)	0.791	b	4	10 (0, 20)	31
CH0228	all	20	24 (21, 28)	0.0313	all	9	15 (12, 19)	9
CH0047	a	11	25 (17, 32)	0.928	a	9	NA	NA*
CH0047	b	7	18 (6, 30)	0.892	b	8	15 (6, 23)	0.851
CH0047	all	18	25 (21, 29)	0.761	all	17	7 (5, 9)	0.185
CH1754	a	88	19 (17, 22)	0.114	a	30	10 (5, 14)	*9.99E-16*
CH1754	b	63	13 (10, 17)	0.0733**	b	15	17 (9, 25)	0.439
CH1754	c	5	11 (4, 18)	72	c	9	21 (11, 31)	0.941
CH1754	d	14	25 (15, 36)	0.416	d	6	16 (8, 24)	0.921
CH1754	e	10	28 (22, 34)	0.439	e	7	19 (12, 26)	0.722
CH1754	all	180	19 (18, 20)	0.206 **	all	67	15 (14, 17)	*0.00256*
CH0275	a	21	12 (8, 17)	0.716				
CH1244	a	35	11 (8, 14)	0.922	a	9	11 (6, 16)	0.277
CH1244	b	9	16 (9, 23)	87				
CH1244	all	44	12 (11, 13)	0.715				
CH0078	a	47	11 (7, 14)	51				

Note: The small p value for CH1754a 5'-half genome sequences was not due to early occurrence of selection or excessive accumulation of mutations. Instead, it was caused by the fact that there were more sequences than expected having two mutations away from the T/F. This could be either due to an early stochastic event or to the fact that this lineage was originated from two very similar T/Fs (1 mutation away from one another). Because these two scenarios cannot be statistically distinguished, CH1754a was defined as a single lineage.

* NA: not applicable due to all identical sequences.

** After removal of APOBEC-biased mutation positions.

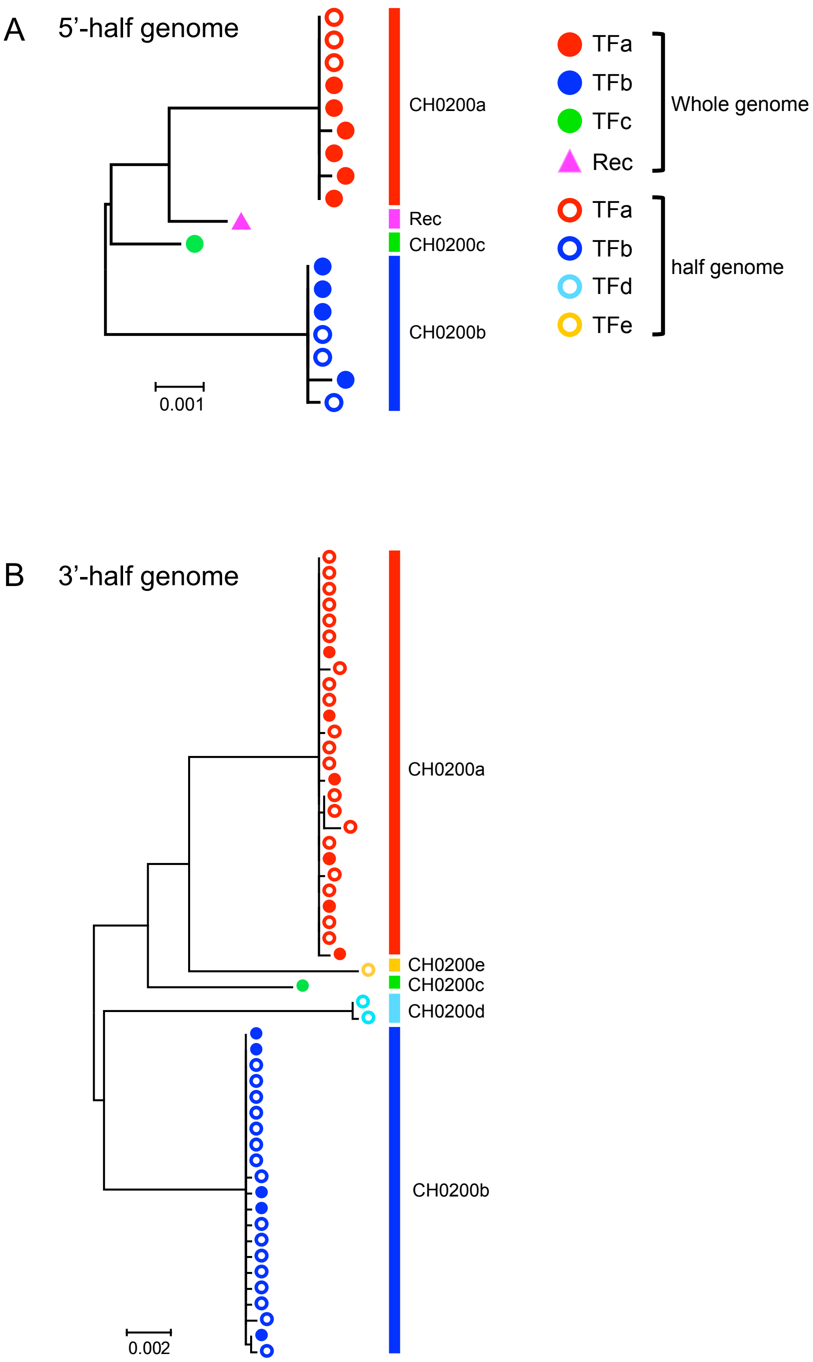

Supplementary Figure 1: Phylogenetic tree analysis of 5'- and 3'-half genome sequences from CH0200. Phylogenetic relationship of 5'-half (A) and 3'-half (B) genome sequences derived from NFLG or half genome sequences were determined using the neighbor-joining method with the K2P substitution model. Sequences derived from T/Fa and T/Fb are indicated in red and blue, respectively. Sequences derived from the NFLG genome are shown in solid symbols and sequences derived from either half-genome are shown in open symbols. T/Fc, T/Fd and T/Fe are indicated by different colors. Recombinants are indicated by triangle.

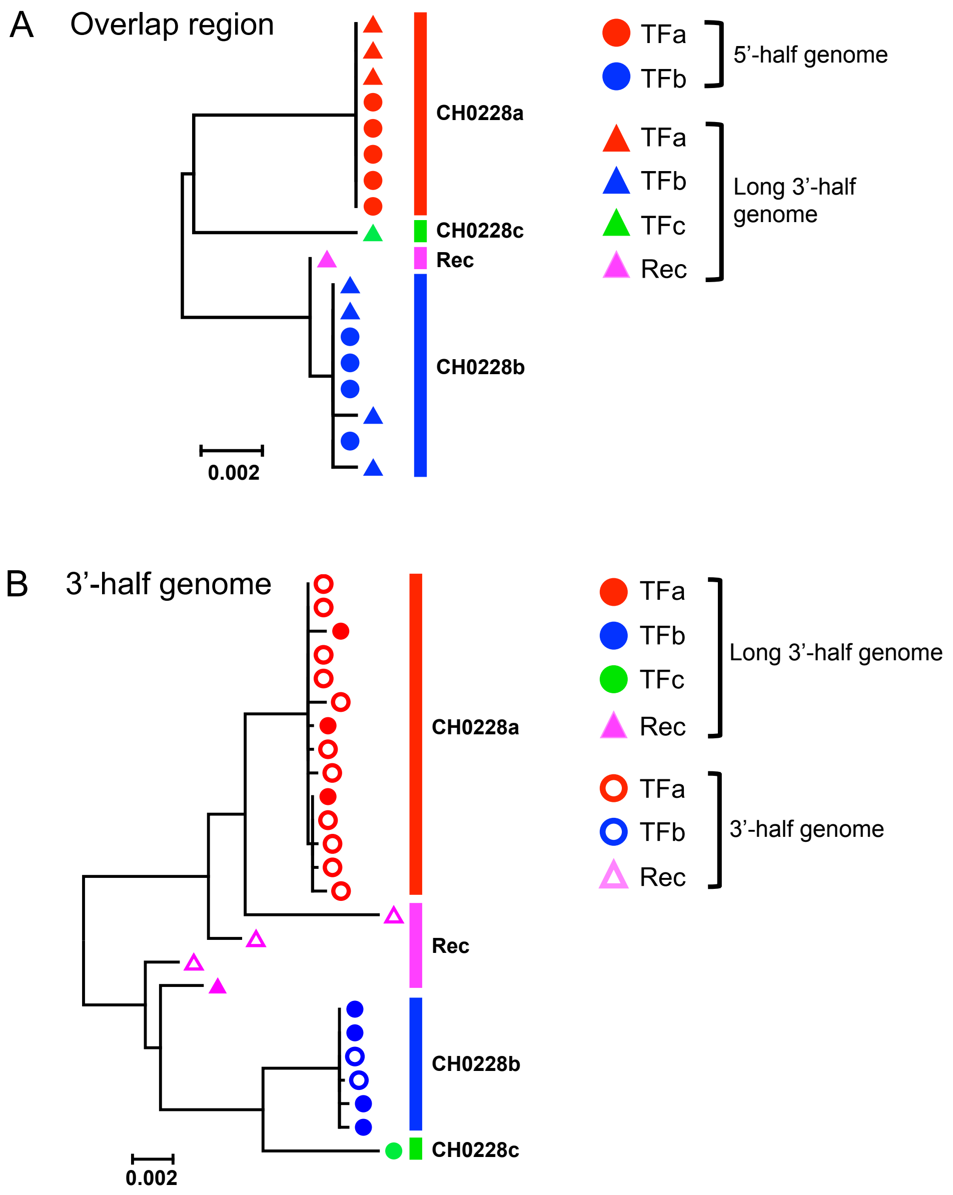

Supplementary Figure 2: Phylogenetic tree analysis of the overlapping region between both half genomes and 3'-half genome sequences from CH0228. (A) The phylogenetic relationship between 5'-half genome sequences and long 3'-half genome sequences at the overlapping region (~1000bp). Sequences derived from T/Fa and T/Fb are indicated in red and blue, respectively. Among them, sequences derived from the 5'-half genome are shown in solid dots and sequences derived from the long 3'-half genome are shown in solid triangles. T/Fc is shown in green triangle. A recombinant sequence is shown as a pink triangle. (B) The phylogenetic relationship between long and short 3'-half genome sequences. Sequences derived from T/Fa and T/Fb are indicated in red and blue, respectively. Among them, sequences derived from the long 3'-half genome are shown in solid dots and sequences derived from the short 3'-half genome are shown in blue dots. T/Fc is shown by green dot. Recombinant sequences are indicated by pink triangle. Phylogenetic trees were constructed using the neighbor-joining method with the K2P substitution model.
